# Earth Worms and the Bacilli of Tuberculosis

**Published:** 1892-10

**Authors:** 


### EARTH-WORMS AND THE BACILLI OF TUBERCULOSIS.

At a meeting of the Academy of Sciences, January 25, 1890, MM. Lortet and Despeignes reported that they had placed earthworms in a vegetable mold, with which they had mixed tuburculous sputum or fragments of tuberculous human lungs. A month later the worms were taken from the earth, opened, the digestinal tube removed, washed with water and alcohol, cut into fragments pulverized, and with this material several guinea pigs were inoculated. These animals fell a prey to acute generalized tuberculosis. sections of different portions of the body of the worms demonstrated that all the tissues, but especially those of the genital organs, contained a great number of the bacilli of tuberculosis mingled with other species of bacteria. The tuberculous microbes gave rise to no special alterations, but were simply infiltrated in little groups in the midst of the cells. It is very probable, therefore, that the worms bring up from the depths of the soil tubercle bacilli, together with earthy fæcal matters forming their dejections. Microscopical examination proves the fact, but inoculations have not succeeded on account of the presence of numerous other species of microbes which are rapidly fatal to guinea-pigs.—*Le Progres Medical, February 6, 1992.*

